# Tidal Modulation of Buoyant Flow and Basal Melt Beneath Petermann Gletscher Ice Shelf, Greenland

**DOI:** 10.1029/2020JC016427

**Published:** 2020-10-13

**Authors:** Peter Washam, Keith W. Nicholls, Andreas Münchow, Laurie Padman

**Affiliations:** ^1^ School of Earth and Atmospheric Sciences Georgia Institute of Technology Atlanta GA USA; ^2^ College of Earth, Ocean, and Environment University of Delaware Newark DE USA; ^3^ British Antarctic Survey Natural Environment Research Council Cambridge UK; ^4^ Earth & Space Research Corvallis OR USA

**Keywords:** ice‐ocean interactions, ice shelves, boundary layer, basal melt, Greenland, glacier

## Abstract

A set of collocated, in situ oceanographic and glaciological measurements from Petermann Gletscher Ice Shelf, Greenland, provides insights into the dynamics of under‐ice flow driving basal melting. At a site 16 km seaward of the grounding line within a longitudinal basal channel, two conductivity‐temperature (CT) sensors beneath the ice base and a phase‐sensitive radar on the ice surface were used to monitor the coupled ice shelf‐ocean system. A 6 month time series spanning 23 August 2015 to 12 February 2016 exhibited two distinct periods of ice‐ocean interactions. Between August and December, radar‐derived basal melt rates featured fortnightly peaks of ∼15 m yr^−1^ which preceded the arrival of cold and fresh pulses in the ocean that had high concentrations of subglacial runoff and glacial meltwater. Estimated current speeds reached 0.20 – 0.40 m s^−1^ during these pulses, consistent with a strengthened meltwater plume from freshwater enrichment. Such signals did not occur between December and February, when ice‐ocean interactions instead varied at principal diurnal and semidiurnal tidal frequencies, and lower melt rates and current speeds prevailed. A combination of estimated current speeds and meltwater concentrations from the two CT sensors yields estimates of subglacial runoff and glacial meltwater volume fluxes that vary between 10 and 80 m^3^ s^−1^ during the ocean pulses. Area‐average upstream ice shelf melt rates from these fluxes are up to 170 m yr^−1^, revealing that these strengthened plumes had already driven their most intense melting before arriving at the study site.

## Key Points

- Concurrent measurements of melt rate and hydrography under Petermann Gletscher Ice Shelf reveal dynamics of ice shelf melting
- Maximum melt rates occur during periods of elevated buoyant under‐ice flow rather than when ocean temperatures are greatest
- Initiation of buoyant flow is modulated at the grounding line by the spring‐neap tidal cycle and seasonality in subglacial runoff

## 1 Introduction

Marine‐terminating outlet glaciers play a key role in the mass balance of the polar ice sheets (e.g., Holland et al., 2008). They transport ice from the center of the ice sheets to their marine margins, where the ice then interacts with warm ocean water and a seasonally warm atmosphere. In an equilibrium state, the ice sheets gain mass through snowfall at the same rate that they lose mass through atmosphere and ocean‐forced melting and calving of their outlet glaciers. Geothermal and frictional heat at the base of these fast‐flowing glaciers also drive melting and remove mass from the ice sheet (Joughin et al., 2003). Studies found that the Greenland (Mouginot et al., 2019) and Antarctic (Shepherd et al., 2018) ice sheets are presently not in equilibrium, but are losing mass, largely due to excess melting and calving at their marine termini (Adusumilli et al., 2020; Enderlin et al., 2014; Pritchard et al., 2012; Rignot & Kanagaratnam, 2006; Smith et al., 2020; van den Broeke et al., 2009). Excess melting erodes the marine termini of the ice sheets' outlet glaciers, which reduces their ability to buttress the seaward flow of ice (e.g., Dupont & Alley, 2005; Thomas & Bentley, 1978). When this occurs, the outlet glaciers accelerate, transport more ice into the ocean, and consequently contribute to global sea level rise. Estimates of ice sheet mass loss reveal that Greenland contributed 13.7 ± 1.1 mm to global sea level rise between 1972 and 2018 (Mouginot et al., 2019), and Antarctica contributed 7.6 ± 3.9 mm between 1992 and 2017 (Shepherd et al., 2018).

Oceanographic and glaciological measurements provide valuable insight into how the ocean and ice sheet interact (e.g., Jenkins et al., 2010). Long‐term glaciological measurements from instruments deployed on the ice sheet surface are scarce; most estimates of ice surface elevation change and velocity come from remote sensing by satellite (Joughin et al., 2010; Rignot et al., 2014; Smith et al., 2020) or aircraft (Dow et al., 2018; Münchow et al., 2014; Rignot et al., 2013). While these remotely sensed measurements reveal large‐scale trends in ice sheet variability, their coarse temporal resolutions do not capture variations across the broad range of time scales over which ocean processes influence ice sheet change. High temporally resolved, long‐term surface‐based measurements of ice thickness from phase‐sensitive radars (e.g., Vaňková et al., 2020) and ice velocity from global positioning systems (e.g., Rosier & Gudmundsson, 2020) have exposed some of these processes on both grounded and floating ice.

Extended time series of ocean conditions near or underneath ice shelves, or at tidewater glacier calving fronts, are also uncommon. Most ocean measurements come from ship‐based conductivity‐temperature‐depth (CTD) surveys in the adjacent water column, such as those provided by Johnson et al. (2011) for Petermann Fjord. However, floating ice shelves and iceberg‐clogged fjords often prevent these CTD surveys from sampling the ocean close to the glacier's grounding line where considerable mass is lost through ocean‐forced ice melt (e.g., Rignot & Jacobs, 2002). Furthermore, heavy sea ice and extreme winter temperatures often restrict ship‐based work to summer and may thus introduce a seasonal bias to these data (Jackson et al., 2014, 2018; Webber et al., 2017).

One approach to collect long‐term ocean time series near glacier grounding lines is to drill a borehole through an ice shelf using pressurized hot water, then deploy instruments through the borehole along a mooring line that is tethered to the ice shelf surface. The mooring line freezes into the ice shelf, which prevents recovery of these instruments. Data acquisition hence relies on communications cables running from the ocean, through the ice shelf, to a data logger on the surface. This approach has been successfully used to collect multiyear ocean time series beneath various Antarctic ice shelves (e.g., Hattermann et al., 2012; Herraiz‐Borreguero et al., 2013; Nicholls & Østerhus, 2004; Stewart et al., 2019). One of the strengths of this approach is that it allows ocean instruments to be deployed in close proximity to the ice base, so that interactions between the ocean and the ice shelf can be monitored.

Very few ice shelves remain in Greenland, and those that do are experiencing ongoing retreat and breakup (Hill et al., 2018). This type of mooring has, therefore, not been utilized as extensively in Greenland as in Antarctica. Indeed, only four sub‐ice‐shelf moorings have been deployed in Greenland: one beneath Nioghalvfjerdsrae Ice Shelf in the northeast (Lindeman et al., 2020) and three beneath Petermann Gletscher Ice Shelf (PGIS) in the north (Münchow et al., 2016; Washam et al., 2018, 2019). We here report on ocean time series data from two SBE 37‐SM Microcat Conductivity‐Temperature (CT) sensors located along one of these mooring lines beneath PGIS within 5 and 25 m of the ice base. In conjunction with the ocean data, we also present ice shelf melt rates acquired using an Autonomous phase‐sensitive Radio‐Echo Sounder (ApRES) collocated on the ice shelf surface. By relating signals in the ocean and melt rate data, we obtain a detailed view of the dynamic interaction between one of Greenland's last remaining ice shelves and the ocean beneath it.

## 2 Petermann Gletscher

**Figure 1:**
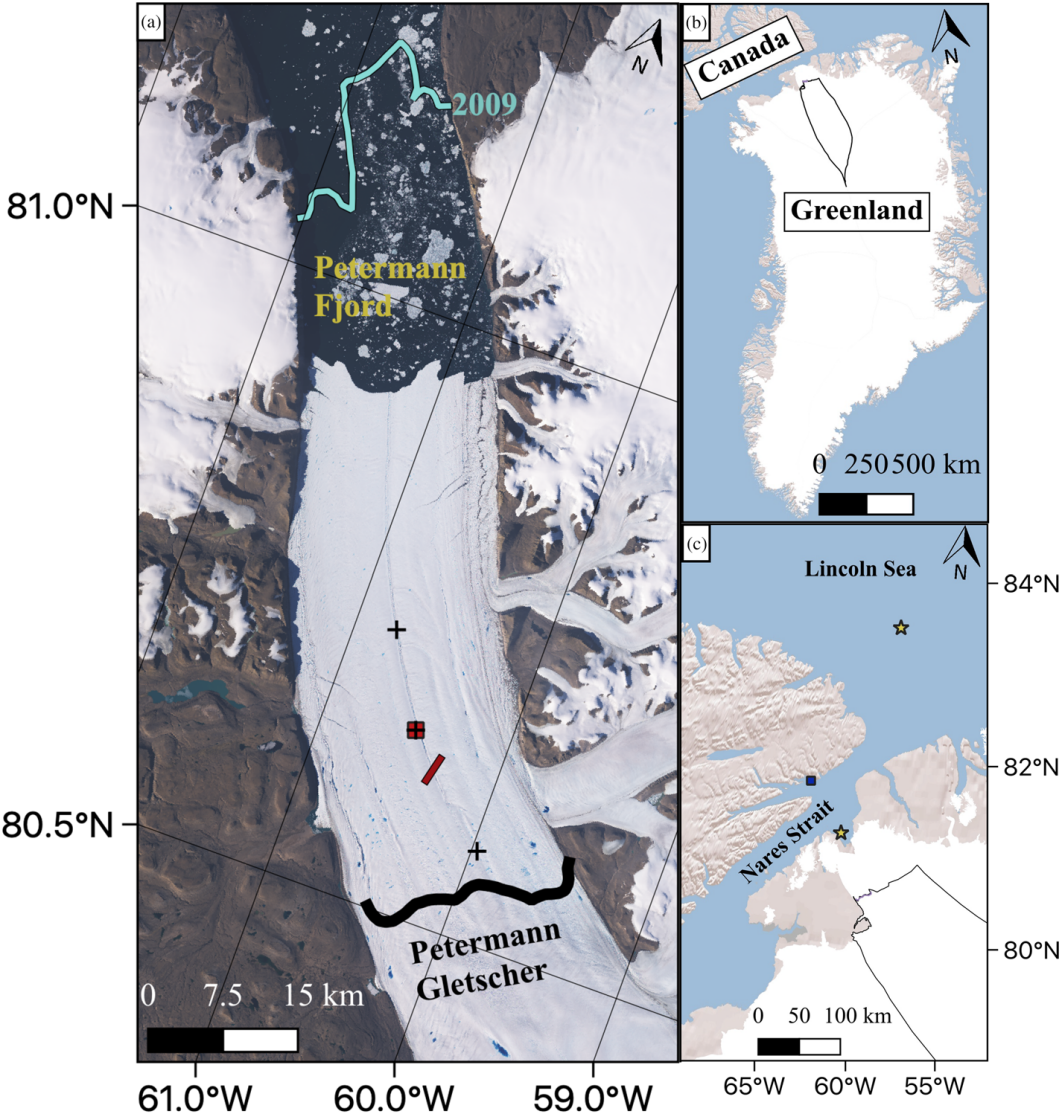
(a) Landsat 8 image from 12 August 2015 displaying Petermann Gletscher Ice Shelf in northern Greenland. The burgundy square indicates the location of our study site, the burgundy line marks a section of a 2015 Operation Icebridge flight line, the black line represents the glacier's grounding line, the aqua line denotes the ice shelf terminus in 2009, and the + markers show the locations of other study sites from Washam et al. (2019). Panels (b) and (c) provide context to the larger geographic region, with the catchment of Petermann Gletscher from Mouginot and Rignot (2019) denoted. The blue square in (c) shows the location of the Discovery Harbor tide gauge, and the yellow stars indicate the locations of bathymetric sills that separate the Lincoln Sea, Nares Strait, and Petermann Fjord.

**Figure 2:**
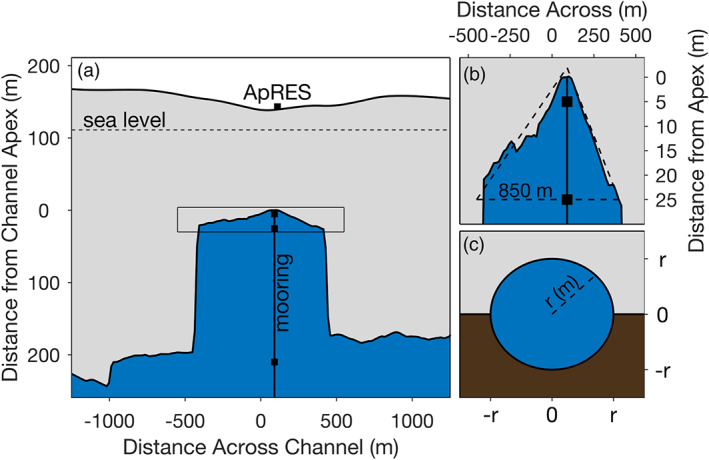
(a) The 5 May 2015 Operation Icebridge airborne ice penetrating radar profile across the PGIS central channel approximately 3.5 km upstream from our study site (see Figure 1 for location), where black squares correspond to the depths of CT sensors along the mooring line and the ApRES location is denoted (10 m lateral distance from mooring), as well as sea level. (b) Upper portion of the channel (represented by box in (a)), where CT sensor depths are indicated, and the dashed triangle represents the area used for flux calculations in section 5.2. (c) Diagram of a model subglacial conduit at the PGIS grounding line that feeds the downstream ice shelf channel.

Petermann Gletscher is the largest outlet glacier in northern Greenland (Mouginot et al., 2019), draining 4% of the ice sheet, by area, across its grounding line into Petermann Fjord (Münchow et al., 2014) (Figure 1) at a nominal speed of 1,200 m yr^−1^ (Münchow et al., 2016). Historically, Petermann Gletscher extended in a 70 – 80 km long ice shelf, but major calving events in 2010 and 2012 shortened it to 50 km (Münchow et al., 2014). The aqua line in Figure 1a represents the ice shelf terminus in 2009, before recent calving events. Prior to calving, the ice shelf thinned by 3 – 5 m yr^−1^ over the 2003–2010 period (Münchow et al., 2014). This likely resulted from stronger ocean‐forced melting along the ice shelf base but could have also arisen from intensified atmospheric‐forced melting of the ice surface during summer (Rignot et al., 2001; Rignot & Steffen, 2008). Relatively warm and saline water of Atlantic origin underlies PGIS (Johnson et al., 2011; Münchow et al., 2016; Washam et al., 2018). This dense Atlantic Water (AW) flows over a 290 m sill from the Lincoln Sea of the Arctic Ocean into Nares Strait, then spills over a 440 m sill from Nares Strait into Petermann Fjord (Jakobsson et al., 2018) (Figure 1c). Once in the fjord, the AW extends from ∼500 m to the seafloor (Johnson et al., 2011), which reaches a depth of up to 1,200 m (Washam et al., 2018). Petermann Gletscher's grounding line rests at 500 – 600 m below sea level (Rignot et al., 2001), within this deep layer of relatively warm AW. The ocean heat in this layer contributes to rapid basal melting near the glacier's grounding line, with annual rates of up to 50 m yr^−1^ (Wilson et al., 2017). Areas of concentrated melting in this region form inverted channels that quickly carve into the ice base, then extend and deepen seaward, parallel to glacier flow (Rignot & Steffen, 2008; Wilson et al., 2017) (Figure 2a). Washam et al. (2019) revealed that the discharge of meltwater across the grounding line played an integral role in modulating basal melt rates at a location in one of these channels. We here extend those analyses by estimating the under‐ice current speeds associated with this buoyancy‐driven flow, as well as those related to tides, as a direct driver of flow and as an impetus of internal wave excitation over topographic slopes. Finally, we combine these estimates with meltwater concentrations to obtain seaward meltwater volume fluxes and area‐averaged melt rates along the upstream section of the ice shelf channel.

## 3 Data and Methods

### 3.1 Field Observations

**Figure 3:**
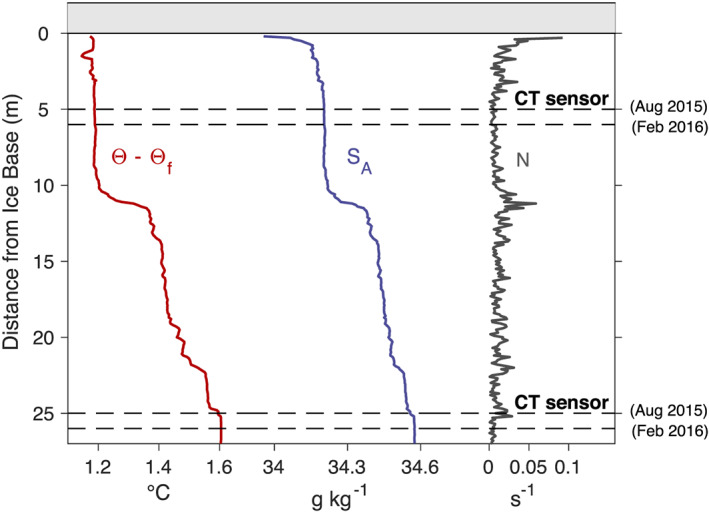
Vertical profiles of conservative temperature above in situ freezing (Θ − Θ_*f*_), absolute salinity (*S*_*A*_), and stratification frequency (*N*) within 25 m of the ice base from a continuous CTD profile collected in August 2015. Overlaid on this is the position of our CT sensors in the water column relative to the ice base at the time of deployment (August 2015) and at the end of the data record (February 2016).

We quantify ice‐ocean interaction beneath PGIS at a site about 16 km from the grounding line in the ice shelf's central basal channel (Figures 1 and 2a). To do this, we exploit collocated, hourly measurements of oceanic properties and local melt rates at the base of the ice shelf. Oceanic properties come from two SBE 37‐SM Microcat CT sensors deployed at 95 and 115 m depth, initially about 5 and 25 m from the ice base (Figure 2b). A CTD profile collected shortly before these sensors were deployed revealed a 10‐m‐thick well‐mixed layer below a sharp pycnocline located within 1 m of the ice‐ocean interface (Figure 3). Ocean properties freshened substantially and cooled slightly upward along this pycnocline to reach a temperature of 1.16°C above the local freezing point at 0.20 m below the ice base, after which they warmed and freshened rapidly due to mixture with the borehole water (contaminated measurements not shown in Figure 3).

The distance between our CT sensors and the ice base increased by 1.00 ± 0.10 m over 6 months due to ocean‐driven basal melting (Figure 3). We monitored these basal melt rates with an ApRES on the ice surface following the methodology of Nicholls et al. (2015). The ApRES was located close to (∼10 m away from) the ocean mooring borehole (Figure 2a) to ensure measured melt rates were consistent with measured and inferred local ocean properties. Melt rate uncertainties are ±0.3 m yr^−1^ and result from related uncertainties in the vertical strain rate (Washam et al., 2019).

Estimates of tidal displacement come from analyses of data recorded every 3 hr between 2003 and 2012 by a Paroscientific pressure sensor that was moored 125 km northwest of PGIS in Discovery Harbor Canada (Münchow & Melling, 2008) (Figure 1c). Predictions based on 38 tidal constituents derived from this time series made at an hourly interval are consistent with simulated tides in Petermann Fjord from a high‐resolution tide model (Padman et al., 2018). Furthermore, comparison between these predictions and short‐term observations of ice shelf tidal displacements from kinematic GPS reveal that the dominant *M*_2_ tide registered similar amplitudes between sites and took only 15 min to travel from Discovery Harbor to PGIS (Münchow et al., 2016). For further details regarding the design of the study site, sensor errors, processing of ApRES data, and validity of these tidal estimates, see Washam et al. (2019).

### 3.2 Estimating Current Speeds With an Ice‐Ocean Boundary Layer Parameterization

We assume that vertical shear between ocean mixed layer currents and the ice shelf base dictates the turbulent vertical flux of ocean heat and salt and therefore the ice shelf melt rate (Jenkins, 1991). As we did not obtain direct measurements of ocean mixed layer currents, we estimate them by inserting our measured temperatures, salinities, and basal melt rates into the boundary layer parameterization described by Holland and Jenkins (1999). This yields a time‐varying ocean current speed, potentially driven by a combination of buoyancy, tides, wind stress acting outside of the sub‐ice shelf cavity, and eddies (e.g., Jenkins, 2011; Mack et al., 2019; Makinson & Nicholls, 1999).

The boundary layer parameterization (Holland & Jenkins, 1999) is as follows:

(1) $$\Theta_{B}=aS_{A_{B}}+b+cp_{B},$$

(2) $$\Delta Q^{T}=Q_{O}^{T}-Q_{I}^{T}=Q_{\text{Latent}}^{T},$$

(3) $$\Delta Q^{S}=Q_{O}^{S}-Q_{I}^{S}=Q_{\text{Fresh}}^{S}.$$

In Equation 1, Θ_*B*_, $S_{A_{B}}$, and *p*_*B*_ represent the respective conservative temperature, absolute salinity, and pressure at the ice‐ocean boundary (conservative temperature and absolute salinity are hereafter referred to as temperature and salinity), and $a=-5.73\times 10^{-2}$°C/(g kg^−1^), $b=9.39\times 10^{-2}$°C, and $c=-7.53\times 10^{-8}$°C Pa^−1^ are considered constants. In this relationship Θ_*B*_ must be at the freezing point. Considerable difficulty surrounds the measurement of these properties because a stratified boundary layer with a virtually unresolvable mm‐thick viscous sublayer separates the ice base from the ocean mixed layer. We therefore solve for the ice‐ocean boundary properties using the above equations. Our method is explained below.

In Equation 2, Δ*Q*^*T*^ represents the divergence of the vertical sensible heat flux at the ice shelf base, which is the difference between the ocean heat flux $Q_{O}^{T}$ into the ice base and the conductive heat flux through the ice shelf $Q_{I}^{T}$. The resultant sensible heat flux equals the latent heat $Q_{\text{Latent}}^{T}$ removed during melting:

(4) $$Q_{\text{Latent}}^{T}=\rho_{i}L_{F}\dot{m},$$

where $\rho_{i}=918$ kg m^−3^ is the density of ice, L ${}_{F}=3.34\times 10^{5}$ J kg^−1^ is the latent heat of fusion, and $\dot{m}$ denotes the measured basal melt rate at our study site ( $\dot{m}>0$ for melting). We use Equation 1 of Jenkins et al. (2010) to compute $Q_{O}^{T}$:

(5) $$Q_{O}^{T}=\rho_{w}c_{p}u_{*}\Gamma_{T}(\Theta -\Theta_{B}),$$

where c_*p*_ = 3,974 J kg^−1^°C^−1^ is the specific heat capacity of cold seawater, and *ρ*_*w*_ and Θ represent the density and temperature of seawater in the ocean mixed layer, measured by our upper CT sensor at approximately 5 m below the ice base (95 m depth). The friction velocity *u*_∗_ is the kinematic stress at the ice‐ocean interface, and the nondimensional turbulent heat transfer coefficient Γ_*T*_ parameterizes the turbulent mixing of ocean heat across the stratified boundary layer and viscous sublayer into contact with the ice base. As will be shown below in Equation 9, *u*_∗_ is a function of ocean mixed layer currents; therefore, the dependence of $Q_{O}^{T}$ on *u*_∗_ establishes our ability to retrieve current speeds from concurrent measurements of hydrographic properties and basal melt rates.

We use Equation 6 of Holland and Jenkins (1999) to compute $Q_{I}^{T}$:

(6) $$Q_{I}^{T}=\rho_{i}c_{p_{i}}\kappa_{i}^{T}{\left.\frac{\delta T_{i}}{\delta z}\right|}_{B},$$

where c ${}_{p_{i}}=2,009$ J kg^−1^°C^−1^ represents the specific heat capacity of the ice shelf and $\kappa_{i}^{T}=1.14\;\times \;10^{-6}$ m^2^ s^−1^ is the molecular thermal diffusivity of the ice shelf. We compute the nonlinear temperature gradient through the ice shelf $\left({\left.\frac{\delta T_{i}}{\delta z}\right|}_{B}\right)$ using Equations 23, 26, and 31 of Holland and Jenkins (1999). This method considers a reduced form of Equation 19 of Holland and Jenkins (1999), where constant vertical advection and diffusion is assumed, as well as a steady state. The steady state assumption follows the justification that for short time scales thickness changes are negligible compared with the total ice shelf thickness. This temperature gradient depends on the melt rate, thickness, and surface temperature of the ice shelf. Here we consider an ice shelf thickness of 100 m, an ice base temperature equal to the time‐varying Θ_*B*_, and an ice surface temperature of −18°C, which represents the median air temperature at our study site from an atmospheric temperature probe (Washam et al., 2019). After solving for Θ_*B*_ (explained below), we find that Q ${}_{I}^{T}$ varies considerably over the range of melt rates considered here (0 to 17 m yr^−1^) but always scales at ∼12% of Q ${}_{O}^{T}$.

In Equation 3, Δ*Q*^*S*^ represents the vertical salt flux divergence at the ice‐ocean interface, where $Q_{O}^{S}$ is the upward salt flux from the ocean and $Q_{I}^{S}$ is the diffusive salt flux through the ice shelf, which we set to 0. Hence, the upward salt flux from the ocean equals the downward freshwater flux required to maintain the ice‐ocean boundary salinity in the presence of melting $Q_{\text{Fresh}}^{S}$:

(7) $$Q_{\text{Fresh}}^{S}=\rho_{i}\dot{m}(S_{A_{B}}-S_{A_{i}}),$$

where $S_{A_{i}}$ represents the salinity of glacial ice that is 0 g kg^−1^. We use Equation 5 of Jenkins et al. (2010) to compute $Q_{O}^{S}$:

(8) $$Q_{O}^{S}=\rho_{w}u_{*}\Gamma_{S}(S_{A}-S_{A_{B}}),$$

where *S*_*A*_ denotes the salinity of seawater approximately 5 m below the ice base in the ocean mixed layer and Γ_*S*_ is the nondimensional salt transfer coefficient, which is typically much smaller than the heat transfer coefficient due to dominant molecular diffusive processes in the viscous sublayer (Steele et al., 1989). Here we relate the heat and salt transfer coefficients with the previously published *u*_∗_Γ_*T*_/*u*_∗_Γ_*S*_ ratio of 25 (Jenkins, 1991; McPhee et al., 1987).

**Figure 4:**
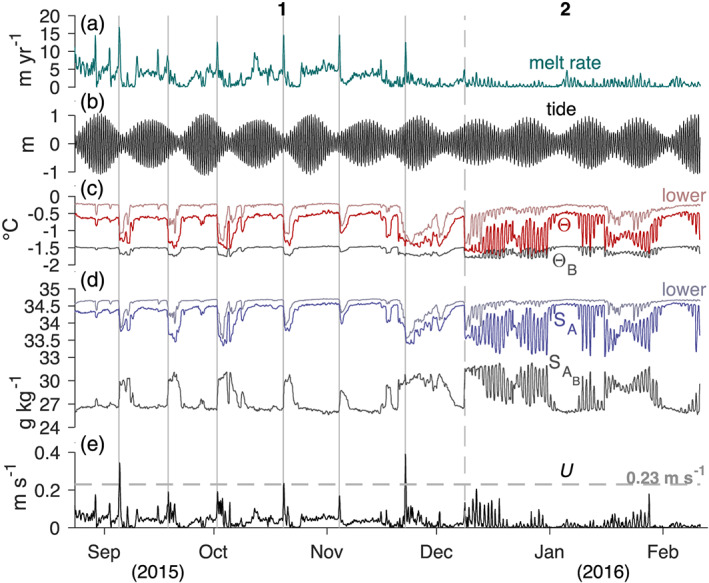
(a) The 23 August 2015 to 11 February 2016 ice shelf basal melt rate, (b) predicted Discovery Harbor tidal fluctuations, ocean mixed layer and ice‐ocean boundary (c) temperature (Θ, Θ_*B*_) and (d) salinity ( $S_{A},S_{A_{B}}$), and (e) estimated ocean mixed layer current speeds from the Holland and Jenkins (1999) parameterization (section 3.2). The additional time series labeled “lower” in (c) and (d) represents temperature and salinity at the lower CT sensor, ∼25 m below the ice base. Solid vertical gray lines underline melt rate peaks that immediately precede cold and fresh ocean meltwater pulses. The vertical dashed gray line partitions Period 1 (23 August to 8 December 2015) from Period 2 (8 December 2015 to 11 February 2016), and the horizontal dashed line in (e) represents the 0.23 m s^−1^ average propagation speed for meltwater pulses (Washam et al., 2019).

A combination of the above equations leads to a quadratic expression for either the combined unknown parameter *u*_∗_Γ_*T*_ or the melt rate that can be solved for without prior knowledge of ice‐ocean interface conditions. We first seek a best fit value for *u*_∗_Γ_*T*_ by tuning this expression to six peaks in our observations of basal melt rates that preceded cold and fresh ocean meltwater pulses (Figure 4). In order to do this, we compute the friction velocity with the quadratic stress formula of McPhee (1979):

(9) $$u_{*}^{2}=C_{D}U^{2},$$

where the mixed layer current speed *U* equals an advective plume speed of 0.23 m s^−1^ that was derived from cross spectral phase lags for the ocean meltwater pulses traveling from another study site near the grounding line, to the site of interest for this study, then to locations further downstream (Washam et al., 2019); see Figure 1a for other study site locations. More specifically, 85% of the variance in the upper ocean CT data at the grounding line site (13 km upstream) correlated with the 115 m CT data from this study site at the principle meltwater pulse frequency of 0.07 cpd (∼14 day period). This signal occurred first at the grounding line site, with a 19° phase or 16 hr time lag, resulting in the 0.23 m s^−1^ advective plume speed. Note that this speed represents an average propagation rate for the meltwater pulses between these two sites, which may differ from the local mixed layer current speed at the the time of their arrival to the site of interest here. As no other information on ocean currents at this site exists, we treat this speed as a best estimate of local conditions and use it to tune the above equations.

The incorporation of an estimate for *U* reduces the unknown parameter for these events to C ${}_{D}^{1/2}\Gamma_{T}$, an expression referred to as the Thermal Stanton number. We use a range of Thermal Stanton numbers to compute melt rates during the six melt rate peaks and select an initial value that produces calculated melt rates closest to our measurements. Differences between calculated and measured melt rates are then attributed to variations in *U*. We address these variations by considering the Thermal Stanton number along with the instantaneous measured melt rate at the arrival time of the six ocean meltwater pulses, then choosing the Thermal Stanton number that produces a median *U* equal to the 0.23 m s^−1^ advective plume speed of Washam et al. (2019). This yields a Thermal Stanton number of 2.18 × 10^−4^, which converts to a Haline Stanton number C ${}_{D}^{1/2}\Gamma_{S}$ of 8.70 × 10^−6^ when we employ a *u*_∗_Γ_*T*_/*u*_∗_Γ_*S*_ ratio of 25 (Jenkins, 1991; McPhee et al., 1987). These values lie toward the lower end or below the two prior sub‐ice shelf observations: C ${}_{D}^{1/2}\Gamma_{T}$ = 0.0011, C ${}_{D}^{1/2}\Gamma_{S}=3.1\times 10^{-5}$ (Ronne Ice Shelf: Jenkins et al., 2010) and C ${}_{D}^{1/2}\Gamma_{TS}=0.87\times 10^{-4}$ (Ross Ice Shelf: Begeman et al., 2018), where Γ_*TS*_ represents an identical transfer coefficient for heat and salt. This is consistent with a level of boundary layer stratification beneath PGIS from high melt rates and subglacial runoff that was not present beneath the Ross and Ronne ice shelves.

After obtaining C ${}_{D}^{1/2}\Gamma_{T}$ and C ${}_{D}^{1/2}\Gamma_{S}$ values from a subset of the data, we employ them to compute a time series of *U* for the entire data record. This is accordingly used to solve for the time‐varying interfacial salinity, then temperature, considering an ice base pressure that decreases over the record with the integrated ApRES melt rate. Finally, these values are returned to the heat (Equations 2 and (4), (5), (6)) and salt (Equations (3), (7), and 8) conservation equations to confirm that computed Δ*Q*^*T*^ and $Q_{O}^{S}$ flux rates equal the respective $Q_{\text{Latent}}^{T}$ and $Q_{\text{Fresh}}^{S}$ rates.

## 4 Results

### 4.1 Melt Rate Response to Ocean Mixed Layer Conditions

Time series of basal melt rates and ocean mixed layer properties depict two distinct periods of ice‐ocean interactions during the 6 months of data (Figure 4). The first 3.5 months (23 August to 8 December 2015) are characterized by melt rates between ∼0 and 17 m yr^−1^ (Figure 4a), which varied in magnitude in relation to the phase of the ∼14 day spring‐neap tidal cycle. Melt rates typically ranged from 2 to 6 m yr^−1^ during spring tidal amplitudes of ∼1 m (Figure 4b), when the ocean mixed layer was relatively warm ( Θ=−0.80 to −0.40°C) and saline ( $S_{A}=34.20$ to 34.60 g kg^−1^), and estimated current speeds were moderate ( U=0.02 to 0.06 m s^−1^) (Figures 4c–4e). During this time the temperature gradient across the boundary layer, that is, the thermal forcing, was large ( $\Theta -\Theta_{B}=0.80$ to 1.00°C); however, the boundary layer was strongly stratified, with calculated interfacial salinities between 26 and 27 g kg^−1^, or 7.20 to 8.60 g kg^−1^ lower than those measured 5 m below in the ocean mixed layer.

As the tidal range declined toward neap conditions, large melt peaks of ∼15 m yr^−1^ occurred, concurrent with short‐duration spikes in estimated current speed of up to 0.40 m s^−1^. These melt peaks preceded cold and fresh pulses in the ocean, where mixed layer hydrographic properties dropped to $\Theta -\Theta_{B}=0.25$°C and $S_{A}=33.50$ g kg^−1^ for several days to over a week. Hydrographic properties at the lower CT sensor also cooled and freshened significantly during these events (Figures 4c and 4d), indicating that the large anomalies reached to at least 25 m below the ice base. Washam et al. (2019) analyzed these ocean pulses and found that they contained up to 4.7 ± 0.2% total freshwater: 2.8 ± 0.1% had derived from meltwater on the grounded glacier that discharged into the ocean across the grounding line (subglacial runoff, denoted SR), and 1.9 ± 0.1% was produced by ocean‐driven melt along the underside of the ice shelf (glacial meltwater, denoted GMW). We infer that the swift estimated current speeds associated with these meltwater pulses led to large shear across the boundary layer, which eroded stratification and raised estimated boundary salinities to ∼30 g kg^−1^. Following this short period of elevated ice‐ocean interaction, currents speeds weakened, and a wake of cold and fresh water was left in the mixed layer, leading to near‐zero melt rates. These conditions persisted until tidal amplitudes increased and melt rate and ocean behavior typical of spring tidal amplitudes returned, except during the last event. Over the 3.5 months, six melt peaks occurred, which were each immediately followed by a meltwater pulse.

After the final meltwater pulse, melt rates reduced to ∼0 to 5 m yr^−1^ for the 8 December 2015 to 11 February 2016 period. Ocean mixed layer properties during this time appeared much different than Period 1, with cooler and fresher conditions ( $\Theta -\Theta_{B}=0.10$ to 0.80°C, $S_{A}=33.20$ to 34.20 g kg^−1^) occurring in spring tides and warmer and saltier ( $\Theta -\Theta_{B}=1.00$°C, $S_{A}=34.50$ g kg^−1^) conditions in neap tides. In addition to this phase switch relative to the spring‐neap tidal cycle, the nature of ice‐ocean variability completely changed during this second part of the record. Melt rates in this period exhibited primarily diurnal fluctuations, with amplitudes of up to 5 m yr^−1^ that generally followed the spring‐neap tidal cycle. Ocean mixed layer conditions also experienced diurnal oscillations that more closely followed the spring‐neap tidal cycle than did the melt rate, with large amplitude variations of 1°C and 1 g kg^−1^ during spring tides and much smaller oscillations of 0.10°C and 0.10 g kg^−1^ during neap tides. Oscillations of smaller amplitude also occurred at the same time in temperature and salinity data 20 m lower in the water column, revealing that these signals were not simply a function of the upper sensor moving in and out of the mixed layer. Estimated mixed layer current speeds during this time also followed the spring‐neap tidal cycle, with average amplitudes ranging from ∼0.05 m s^−1^ during spring tides to <0.01 m s^−1^ during neap tides. These current fluctuations notably affected stratification, with estimated salinity differences across the boundary layer ranging from 1.00 to 7.50 g kg^−1^ during large amplitudes and 8.40 to 8.80 g kg^−1^ during low amplitudes. Note that the current speeds estimated for Period 2 are higher than simulated barotropic (depth‐averaged) tidal current speeds at our site (Padman et al., 2018), which reach only 0.01 m s^−1^ during spring tides and are negligible during neap tides. A possible mechanism to explain this discrepancy in speed is described in section 5.1.

The above estimates of current speed and interfacial temperature and salinity hinge on the choice of constant Thermal and Haline Stanton numbers for the full data record. While the lack of measured current speeds and upper ocean hydrographic vertical resolution necessitated this assumption, the implementation of constant turbulent heat and salt transfer coefficients during variable melt and mixed layer conditions could have led to inconsistencies in the estimated variables. Stanton numbers are likely most accurate during the melt rate peaks at the arrival time of meltwater pulses, as these events were used to tune the boundary layer parameterization equations, but the efficiency of turbulent mixing of heat and salt across the boundary layer varies with the degree of upper ocean stratification. In periods of high thermal forcing and low melting such as Period 1 background conditions, increased stratification would potentially reduce the efficiency of turbulent mixing. This would lower the Stanton numbers relative to those estimated here, which would require a combination of higher current speeds and higher interfacial salinities (weaker stratification) to drive the observed melt rates. This could indicate that our estimated currents and interfacial salinities are biased low and our interfacial temperatures are biased high during this section of the record. In contrast, in periods of colder and fresher ocean conditions and low melting such as during Period 2 spring tides, decreased stratification would potentially increase the efficiency of turbulent mixing. This would raise the Stanton numbers relative to those estimated here, which would require a combination of lower current speeds and higher interfacial salinities (weaker stratification) to drive the observed melt rates. This could indicate that our estimated currents and interfacial temperatures are biased high and our interfacial salinities are biased low during this section of the record.

**Figure 5:**
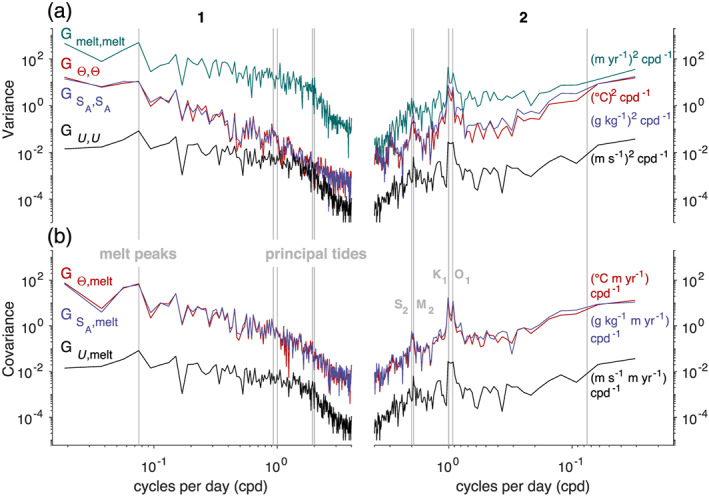
(a) Autopower spectra for melt rate and ocean mixed layer Θ, *S*_*A*_, and estimated *U* data from Period 1 (left panel) and Period 2 (right panel). (b) Cross‐power spectra between melt rate and Θ, *S*_*A*_, and *U* data. Vertical gray lines indicate frequencies associated with principal tidal constituents ( $K_{1}=1.01$ cpd, $O_{1}=0.93$ cpd, $S_{2}=2.00$ cpd, $M_{2}=1.93$ cpd) and melt rate peaks (0.07 cpd, ∼14 day period) from Figure 4.

Autopower spectra of the data from these two periods reveal the distribution of variability at discrete time scales in the ice shelf basal melt rate, ocean mixed layer hydrographic properties, and estimated current speeds (Figure 5a). When compared, the melt rate and ocean spectra from Period 1 display higher variability at frequencies lower than one cycle per day (cpd) than during Period 2. This is highlighted by the spike in all four spectra at 0.07 cpd (∼14 day period), the dominant frequency of the melt rate peaks and ocean meltwater pulses in Period 1 (Figure 4). This signal is not present during Period 2; instead, spectral peaks appear in the data at the principal diurnal ( $K_{1}=1.01$ cpd, $O_{1}=0.93$ cpd) and semidiurnal ( $S_{2}=2.00$ cpd, $M_{2}=1.93$ cpd) tidal frequencies. While these frequencies dominate variability during Period 2, melt rate spectra in Period 1 register comparable diurnal levels and higher semidiurnal levels than Period 2. However, elevated Period 1 background melt rate variance masks these signals, which suggests different regimes between periods. The ocean spectra corroborate this, as diurnal and semidiurnal levels are much lower during Period 1 than Period 2.

Cross‐power spectra confirm that the basal melt rate did indeed covary with the mixed layer hydrographic properties (Figure 5b), as spectral peaks are visible at the frequencies of interest from both periods. The melt rate also covaried with the estimated current, which is unsurprising as this variable was derived from the melt rate and hydrographic property time series. Note that the cross‐power spectra between the melt rate and Θ, and the melt rate and *S*_*A*_ closely align with one another. This is consistent with highly correlated variability of Θ with *S*_*A*_ in the mixed layer.

## 5 Discussion

### 5.1 Influence of Buoyancy Forcing and Tides on Under‐Ice Currents and Melting

**Figure 6:**
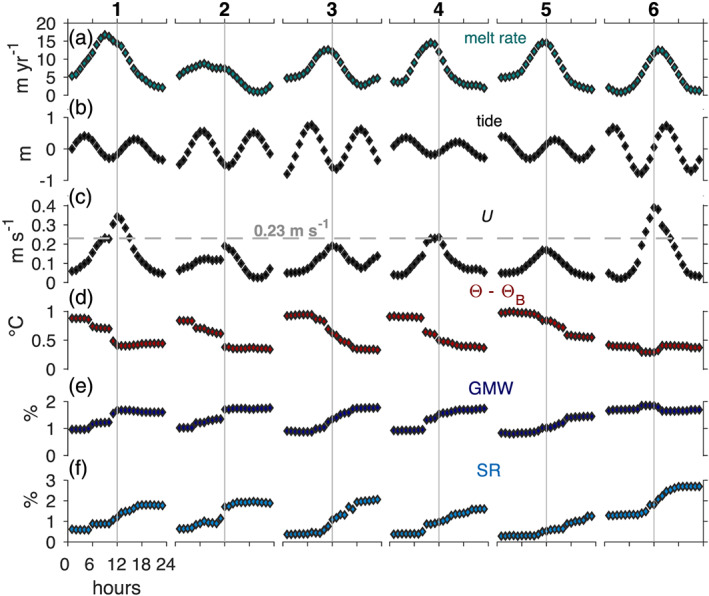
(a) The 24 hr structure of the six melt rate peaks underlined in gray in Figure 4. (b) Predicted Discovery Harbor tidal fluctuations. (c) Estimated ocean mixed layer current speeds for these melt rates. (d) Ocean mixed layer thermal forcing, (e) glacial meltwater (GMW), and (f) subglacial runoff (SR) concentrations at 95 m depth. Figure panels center on the local maxima for estimated current speeds, which are marked by vertical gray lines. For details regarding the calculation of GMW and SR concentrations see Washam et al. (2019).

The lack of semidiurnal and diurnal tidal variations in estimated ocean currents during Period 1 suggests that some other component dominated flow beneath the ice shelf during this time. The higher speeds in Period 1 compared with Period 2 further demonstrate that this other component contributed significantly to under‐ice currents and the resultant ocean‐forced melt (Figure 4). Washam et al. (2019) attributed this component of flow to buoyancy forcing imparted by high concentrations of fresh SR and GMW in the ocean mixed layer during this time. The effect of buoyancy forcing on current speeds and ocean‐driven melt is clearest during the six cold and fresh ocean pulses in Period 1 (Figure 4). Washam et al. (2019) show that discharge of SR across the grounding line stimulates these pulses and strengthens the buoyant meltwater plume in this region. The plumes then continue to accumulate freshwater as they travel seaward through input of GMW into the water column from melting. At our site, the spikes in current speeds and melt rates preceding the arrival of these pulses reveal that this additional buoyancy from SR and GMW greatly impacts ice‐ocean interactions. To investigate the dynamics of how accelerated buoyancy‐driven currents induced stronger ocean‐forced melt, we focus on 1 day snapshots of the melt rate, tide, current speed, mixed layer thermal forcing, and GMW and SR concentration during the six meltwater pulses from Period 1 (Figure 6).

In each event, melt rates (Figure 6a) began to rapidly rise in phase with the estimated current speed (Figure 6c) about 8 hr before the local maximum melt rate, which typically occurred around low tide (Figure 6b). Mixed layer thermal forcing (Figure 6d) dropped over this time due to increased concentrations of cold (and fresh) GMW, as well as fresh SR (Figures 6e and 6f). These colder and fresher conditions began to lower the local melt rate even though currents continued to rise to a maximum that aligned with the arrival of the meltwater pulse, when mixed layer thermal forcing registered about 0.5°C lower than 12 hr previously, and GMW and SR concentrations significantly increased. After the front of this strengthened plume advected past our site, currents weakened, and a wake of cold and fresh water remained in the mixed layer, which led to low melt rates.

This pattern of buoyancy‐enhanced melt held true for five of the six meltwater pulses; however, the sixth pulse exhibited a different structure, as thermal forcing was already low, meltwater concentrations were high, and the peak current speed preceded the peak melt rate. This event marked a maximum current speed of 0.40 m s^−1^ and occurred as tidal amplitudes were increasing, rather than decreasing or at a minimum (Figure 4).

**Figure 7:**
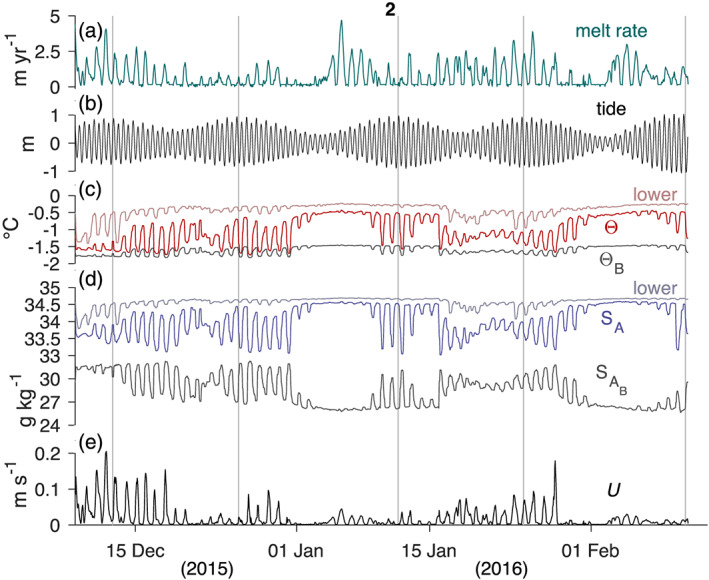
As in Figure 4, except only for Period 2 (8 December 2015 to 11 February 2016). Vertical gray lines underline the maximum tidal range during each spring‐neap cycle.

Washam et al. (2019) noted that the concentration of fresh SR and GMW during background or nonpulsed conditions gradually declined from August to December 2015, which weakened buoyant flow over this time. Our estimated current speeds during these background hydrographic conditions ( $\Theta -\Theta_{B}=0.80$ to 1.00°C, $S_{A}=34.20$ to 34.60 g kg^−1^) decreased from ∼0.06 m s^−1^ in late August to ∼0.03 m s^−1^ in December (Figures 4c –4e). Eventually these currents abated to a point where they no longer dominated flow, and regimes shifted suddenly on 8 December 2015, when melt rates and ocean properties began to oscillate at diurnal tidal periods (Figures 4, 5, and 7).

In Period 2 the ocean mixed layer oscillations at our study site are diurnal (Figures 7c–7e) even though semidiurnal tides dominate in pressure (Figure 7b), current speeds in the adjacent Nares Strait (Davis et al., 2019; Münchow & Melling, 2008), and the vertical motion of the ice shelf itself (Münchow et al., 2016). One would expect the opposite to occur during weak or absent background flow, as vertical mixing is a function of speed which peaks twice during an oscillating current. Diurnal tidal oscillations in such an environment produce semidiurnal signals in current speed and semidiurnal tides produce a 4 cpd signal in speed. Since we instead observe a dominance of diurnal energy in Period 2, we propose that an internal wave process is responsible for the observed diurnal mixed layer variability during this time.

Freely propagating internal waves exist only for periods less than the inertial period $\frac{2\pi }{f}$ (Gill, 1982), which in our study area equals about 12.16 hr. Petermann Fjord thus resides poleward of the so‐called “critical latitude” and propagating internal tidal waves decay exponentially away from their generation site for all diurnal and most semidiurnal tides (Makinson et al., 2006; Robertson et al., 2017). Internal tidal oscillations at subinertial frequencies can therefore only reach the ice shelf as baroclinic Kelvin or topographic Rossby waves that require a vertical wall or sloping bottom, respectively (Gill, 1982). While Sun et al. (2019) attributed observed subinertial diurnal oscillations in the basal melt rate along the grounding line of the broad Roi Baudouin Ice Shelf, Antarctica to topographic Rossby waves, we posit that the steep and narrow Petermann Fjord may more likely facilitate baroclinic Kelvin waves. We speculate that the diurnal baroclinic waves are generated by the diurnal barotropic tide traveling across the fjord sill, approximately 65 km seaward of our study site. When early‐winter deepening of the upper mixed layer pycnocline in this uncovered portion of the fjord allowed this interface to reach the approximate depth range of our sensors, these waves could then travel along the interface and drive the observed ice‐ocean interactions at our site. Indeed, an 18 hr ship‐based survey of tidal currents at the fjord sill during summer revealed an intensified flow focused at the approximate depth of the upper mixed layer pycnocline, although it was assumed to be a result of the semidiurnal tide (Johnson et al., 2011; their Figure 6). The phase speed of a mode‐1 baroclinic Kelvin wave is $c=\frac{ND}{\pi }$, which we estimate from the CTD profile taken beneath the ice shelf prior to mooring deployment (Figure 3). Considering a typical value of N=0.025 s^−1^ within D=25 m of the ice base produces c=0.20 m s^−1^. A wave traveling at this speed would take 3.5 days to reach our study site from the fjord sill, which is consistent with observations in Period 2 when the largest fluctuations in the ice‐ocean system generally lagged maximum tidal amplitudes (Figure 7).

The steeply sloping walls of Petermann Fjord (500 m drop over 1,000 m distance) prevent linear Rossby wave theory from applying. Ignoring this limitation, we estimate the along‐slope phase velocity of a (short) diurnal topographic Rossby wave in a two‐layer ocean with an upper layer D=25 m to be about 0.002 m s^−1^ (dispersion relation from Rhines, 1970). This propagation speed is 2 orders of magnitude smaller than the corresponding Kelvin wave speed and thus could not explain our observations.

### 5.2 Estimates of Meltwater Fluxes

We interpret the behavior of estimated currents in Period 1 as a persistent, seaward flowing meltwater plume that periodically accelerated when a large pulse of SR discharged across the grounding line. This interpretation is supported by phase‐lagged cross correlations over this time period between three mooring sites, which showed that ocean mixed layer signals occurred first near the grounding line, then at the site of interest here, and finally at a location further seaward (Washam et al., 2019). During these advective pulses, the GMW and SR concentrations at our CT sensor 25 m from the ice base closely resembled those within 5 m of the ice (not shown). This was not the case for background hydrographic conditions, which indicates that the accelerated plume thickened the upper mixed layer to at least the depth of this lower sensor. The increase in mixed layer meltwater content and current speed suggests that a considerable amount of meltwater advected past our study site during these pulses. We estimate meltwater volume fluxes (*Q*_*GMW*_, *Q*_*SR*_) past our site in Period 1 by combining the meltwater fractions (GMW, SR) from our two CT sensors with inferred current speeds, then considering the cross‐sectional area of the central basal channel of PGIS through which this water flows.

The best estimate of across‐channel geometry comes from an ice penetrating radar profile acquired on 5 May 2015 approximately 3.5 km upstream of our study site (Figure 2a). The upper section of the channel was triangular, approximately 25 m tall by 850 m wide (Figure 2b); we use this to estimate a cross‐sectional area of 10,625 m^2^. For GMW and SR content, we take three approaches to provide estimates with higher and lower bounds. First, we linearly interpolate fractions between our sensor at 5 m from the ice and our sensor at 25 m from the ice, then set fractions in the upper 5 m of the water column to the value at the upper sensor. This approach likely underestimates meltwater content in the upper 25 m of the water column, as a continuous profile through this region from August 2015 revealed a 10 m thick layer of nearly constant concentrations underlain by linearly decreasing values (Washam et al., 2019; Figure 4b). Accordingly, in the second approach we estimate higher bounds on meltwater content by setting the upper 10 m of the water column to the fractions at our sensor 5 m from the ice, then linearly interpolating downward to our sensor at 25 m from the ice. Finally, we obtain lower bounds for meltwater content estimates by setting the upper 5 m of the water column to the fractions at our upper sensor, then setting the lower 20 m of the water column to the lower sensor fractions. We then assume that our meltwater content estimates are representative of the entire upper water column within 25 m of the channel apex at any point in time, and that this area flows uniformly seaward at the speeds shown in Figure 4e. These three approaches yield meltwater volume fluxes with ranges in the 90th percentile of 0.2 to 1.2 and 0.0 to 1.8 m^3^ s^−1^ for GMW and SR, respectively.

**Figure 8:**
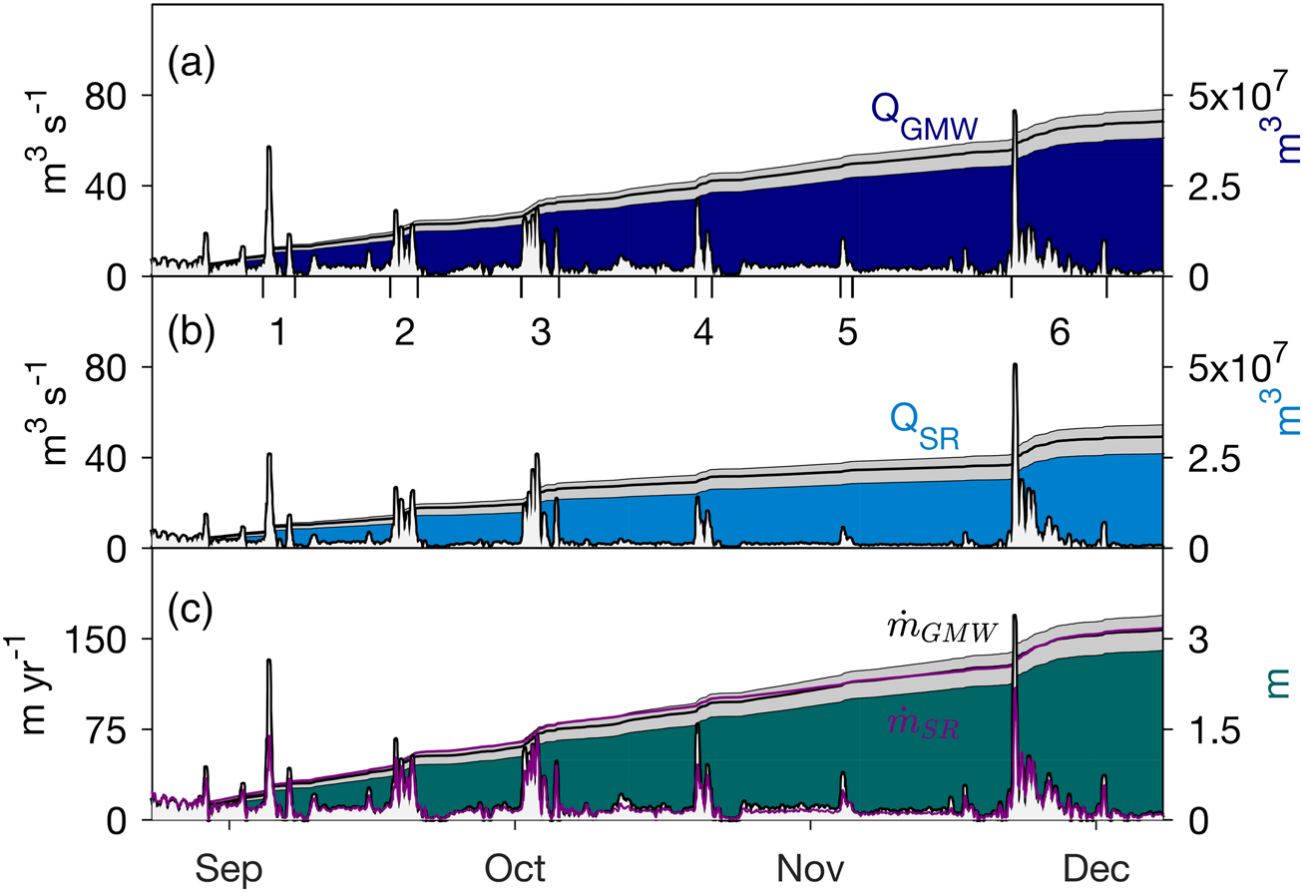
Estimates of (a) GMW and (b) SR flux past our study site during Period 1 (left axis: instantaneous rate, right axis: total volume). (c) Bulk melt rate over the 16 km upstream section of ice shelf channel between our site and the grounding line, where the black line is computed using GMW fluxes and the purple line is computed using SR fluxes (left axis: instantaneous rate, right axis: accumulated melt). Gray shading behind total volumes and accumulated melt indicate upper and lower bounds on estimates from respective ranges in instantaneous flux rates.

We estimate that 4.3 ± 0.4 × 10^7^ m^3^ of GMW and 3.0 ± 0.4 × 10^7^ m^3^ of SR advected past our study site over the 106 days between 23 August and 8 December 2015 (Figures 8a and 8b). Of this total volume, 46 ± 4% of the GMW and 61 ± 7% of the SR was transported over 23 days in the six large pulses. During these pulses, instantaneous flux rates rose considerably over background conditions, with a maximum of 73 ± 2 and 81 ± 1 m^3^ s^−1^ on 22 November in the final pulse for *Q*_GMW_ and *Q*_SR_, respectively. This event not only registered the highest flux rate, but also the longest duration of elevated flux, transporting 17 ± 1% of the total GMW and 24 ± 3% of the total SR past our site over 10 days.

The impact of these swift meltwater pulses on ocean‐driven melting is clear in the ∼12 hr of elevated local melt rates surrounding their arrival at our study site (Figure 6); however, the considerable volume of freshwater transported by them is the result of integrated melting upstream. We estimate the bulk melt rate ( ${\dot{m}}_{\text{GMW}}$) along the upper section of the ice shelf basal channel between our site and the grounding line by dividing *Q*_*GMW*_ by an 850 m × 16,000 m area, the product of the channel width near our site and distance to the grounding line (Figures 1 and 2). This estimate assumes the ice shelf channel width does not change between our site and the grounding line and that *Q*_*GMW*_ results only from melting within the channel. Upper and lower bounds on these melt rates result from associated bounds on *Q*_*GMW*_ flux rates.

Bulk upstream melt rates using this method (Figure 8c) vary considerably over the record, with minimum values of 0 m yr^−1^, maxima of 40 to 170 m yr^−1^, and a median of 8 m yr^−1^. Overall, these rates far exceed those measured locally at our study site, which have a median of 3 m yr^−1^ and maxima of 10 to 17 m yr^−1^. Thus, the persistent GMW flux past our study site indicates that more intense melting occurred upstream during both background and pulsed conditions. Meltwater pulses impacted upstream melting differently than at the local study site, as they drove heightened rates for most of their duration (Figure 8c) instead of a single peak in melting followed by near‐zero conditions (Figure 4a). This is most noticeable during the sixth pulse, which marked the maximum melt rate and was responsible for driving 0.5 ± 0.2 m of melt along the 16,000 m length and 850 m width of the channel over a period of 10 days. We attribute the differing upstream versus local melt rate behavior to a balance between the speed and ocean heat content in the evolving meltwater plume, which ultimately dictates the vertical ocean heat flux into the ice base. As the meltwater plume traveled along the ice base between the grounding line and our study site, it drove melting and cooled, producing a mixture of GMW that depended upon the degree of melting. In the six large meltwater pulses, the upstream melting was strong enough to remove much of the heat from the ocean before the pulse arrived at our study site (Figure 4). Although some of these plumes exhibited significant current speeds at our local study site following the maxima associated with their propagating fronts, the decreased ocean heat led to a low heat flux and melt rate. This would not be the case closer to the grounding line, where less ocean heat would have been removed through melting, and the elevated buoyant currents could still entrain warm seawater and drive a considerable ocean heat flux into the ice base.

We estimate that the total upstream portion of the channel melted by 3.1 ± 0.3 m over 106 days, which is three times higher than the 1.0 ± 0.1 m of melt at our study site over this time. While this crude estimate of melting does not resolve along‐channel variability, it does reveal that stronger melting occurred beneath the ice shelf near the grounding line. This is consistent with remotely sensed melt rates of PGIS that indicate this to be the region of maximum melt (Münchow et al., 2014; Rignot & Steffen, 2008; Wilson et al., 2017), and contemporaneous sub‐ice shelf CTD profiles that show undiluted warm and saline Atlantic Water (AW) that was 2.5°C above in situ freezing at the depth where the ice shelf basal channel becomes grounded (Washam et al., 2018; Figure 7b). Furthermore, the 1:3 ratio in local versus upstream melt is consistent with simulations of buoyancy‐driven melt beneath PGIS, considering an AW temperature at the grounding line of 2.1°C above in situ freezing (Cai et al., 2017).

The coupled relationship between SR flux rates and bulk melt rates (from GMW fluxes) indicates that the discharge of SR across the grounding line placed a first order control on the strength of channelized melting downstream. Previous modeling studies derived an empirical relationship to relate the maximum melt rate at a tidewater glacier (Xu et al., 2013) as well as the maximum and area‐averaged melt rate beneath an ice shelf (Cai et al., 2017) to the SR flux. This was achieved by using a least squares fit to express the simulated melt rate as a function of the SR flux. While we expect melting to still occur without this additional forcing, we use Equation 1 of Xu et al. (2013) and Cai et al. (2017) to determine the degree with which bulk melt rate variability can be explained by variations in our estimated SR flux:

(10) $${\dot{m}}_{\text{SR}}=(Aq_{\text{SR}}^{\alpha }+B){\left(\Theta_{AW}-\Theta_{f}\right)}^{\beta }.$$

**Figure 9:**
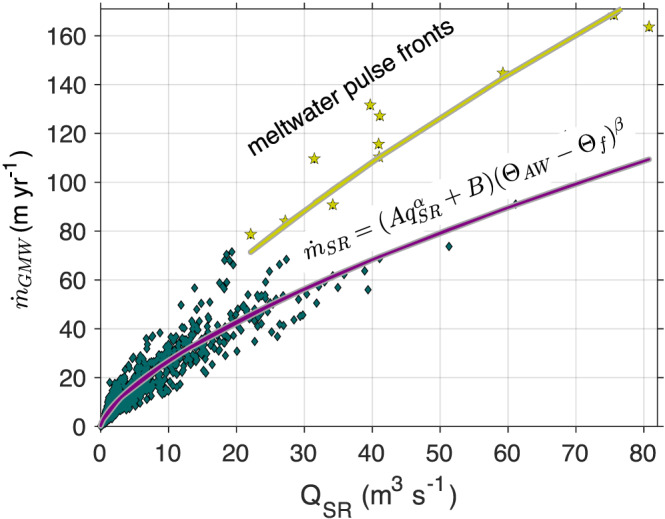
Bulk upstream melt rates from GMW flux versus SR flux, with the best fit of Equation 10 for all data overlaid in purple and for meltwater pulse fronts with melt rates >75 m yr^−1^ in yellow.

In Equation 10, ${\dot{m}}_{\text{SR}}$ represents the simulated bulk melt rate along the 16 km section of ice shelf channel as a function of the time‐varying SR discharge speed across the grounding line *q*_SR_ and the AW temperature above freezing in this region Θ_*AW*_ − Θ_*f*_ = 2.5°C; the remaining parameters are constants. The SR discharge speed is tuned by dividing *Q*_SR_ by various hypothetical two‐dimensional, circular subglacial conduit areas through which this freshwater flowed as it crossed the grounding line (Figure 2c), and adjusting the remaining constants. After considering a range of conduit sizes and constant values, we find a radius of 15 m and constants of A=0.0016, α=0.67, B=0.01, and β=1.11 provide the best fit (Figure 9), with RMS residuals between ${\dot{m}}_{\text{GMW}}$ and ${\dot{m}}_{\text{SR}}=4.6\;\pm$ 0.2 m yr^−1^.

Overall, melt rates derived from SR fluxes show good agreement with those estimated from GMW fluxes (Figures 8 and 9), with the ${\dot{m}}_{\text{SR}}$ accumulated melt over the record lying within the range for ${\dot{m}}_{\text{GMW}}$. Substantial discrepancies only occur during melt rates >75 m yr^−1^ caused by the propagating fronts of the meltwater pulses, when ${\dot{m}}_{\text{SR}}$ register as low as one half of ${\dot{m}}_{\text{GMW}}$. Note that this is the case even though our melt rate power dependency on SR flux α=0.67 exceeds prior published values from modeling exercises that consider large flux rates: 0.33 (Jenkins, 2011), 0.54 (Xu et al., 2013), and 0.56 (Cai et al., 2017). In order to fit ${\dot{m}}_{\text{SR}}$ to ${\dot{m}}_{\text{GMW}}$ during these events a larger *α* of 0.70 must be used (Figure 9). This model therefore suggests that melt rates in the central channel of PGIS are more sensitive to SR flux rates than previous theoretical studies indicated.

### 5.3 Timing of Meltwater Pulses

All six meltwater pulses during Period 1 arrived at our downstream study site when tidal amplitudes were low (Figure 4), indicating that the spring‐neap tidal cycle controlled the stimulation and evolution of buoyant flow beneath the ice shelf. One oceanic process that could have modulated the evolution of the meltwater plume is a tidal front located in the narrow ocean cavity near the grounding line (Holland, 2008; MacAyeal, 1984). In the presence of such a front, turbulent mixing between tidal currents and the ice and seafloor completely erodes stratification to create a fully mixed water column (Fearnhead, 1975; Simpson & Hunter, 1974) that prevents the downstream transport of fresh water. As tidal amplitudes recede toward neap conditions, the associated currents weaken and so does this barrier. However, estimates of the tidal current speeds necessary to trap the meltwater pulses at the grounding line reveal that they would need to be unrealistically high, on the order of tens to a hundred m s^−1^ (Appendix A). Therefore, we conclude that tidal mixing near the grounding line cannot explain the observed spring‐neap control on SR discharge.

An alternate hypothesis is that tidally induced grounding line flexure and migration modulates subglacial pressure gradients, thus controlling the timing of subglacial outflow. Prior modeling efforts have sought to address this problem with a simplified 2‐D viscoelastic or purely elastic floating ice shelf that transitions to grounded ice superimposed on an elastic bed (Sayag & Worster, 2013; Walker et al., 2013). If the grounding line's horizontal position is kept fixed, then vertical tidal displacements cause seawater to be pumped upstream during low tides and outflow of fresh or brackish water into the ocean during high tides (Walker et al., 2013). Conversely, if the grounding line is allowed to migrate horizontally with the tide along a shallow sloping bed, such as Hogg et al. (2016) suggest is the case for a portion of the PGIS grounding line encompassing the central channel, then upstream subglacial water moves back and forth with the tide but never crosses the grounding line (Sayag & Worster, 2013). This is due to enhanced subglacial pressure at the grounding line relative to upstream, which creates a hydraulic barrier.

Several studies suggest that a large subglacial canyon upstream of PGIS feeds the central ice shelf channel (Bamber et al., 2013; Bell et al., 2014; Chambers et al., 2019). Whether or not this feature manifests at the grounding line as a conduit etched into the sediment, ice, or both is unknown. Neither of the aforementioned idealized models contain the details of how a subglacial conduit might respond to tidally induced grounding line variability. Nevertheless, they both show that grounding line motion and flexure scales with the tidal range, and that the effect on subglacial water movement is greatest when the tidal range is largest. If we apply the Sayag and Worster (2013) model to the PGIS grounding zone, then as tidal flexure at the grounding line diminishes into neap tides, the associated hydraulic barrier preventing upstream freshwater from discharging into the ocean also weakens. Thus, the freshwater that stalled at the grounding line during spring tides would overcome this weakened barrier and escape into the ocean to initiate a seaward flowing buoyant meltwater plume near neap tides.

In the Walker et al. (2013) scenario, tidal pumping of freshwater and seawater across the grounding line would be most vigorous during spring tides. Our estimates of SR discharge speeds across the grounding line (*q*_*SR*_, Equation 10) vary from 0.01 to 0.12 m s^−1^ for meltwater pulses, with only the sixth pulse registering above 0.06 m s^−1^. Given these relatively low current speeds, we suggest that while large tidal amplitudes persisted, freshwater and seawater can be pumped back and forth across the grounding line with the tide at a rate that prevents meaningful seaward propagation. Additionally, this back‐and‐forth mechanical movement across the grounding line would serve to mix the water masses in the narrow water column, thus reducing buoyancy forcing relative to a monotonic outflow of fresh water. As tidal amplitudes receded toward neap conditions, the associated pumping would weaken to a point when the meltwater pulse could escape this region, then rise along the sloping ice base as a buoyant plume, accumulate more freshwater through melting, and arrive at our downstream study site.

The large SR fluxes associated with these pulses reveal that when each did escape the grounding line, it evacuated a considerable amount of subglacial water. Following this evacuation, the upstream reservoir likely refilled over the following spring tidal cycle, then discharged again around neap tide. We posit that the pulsed behavior of these events is a function of a diminishing supply of freshwater to the glacier bed as remnant surface melt from the previous summer became less prevalent (Washam et al., 2019). Assuming that these pulses largely consisted of surface meltwater, our data suggest a 3 month lag between the end of surface melting in early September when air temperatures dropped below freezing (Washam et al., 2019; Figure 3) and the discharge of most of this water into the ocean. Finally, after the sixth meltwater pulse, this source became low enough that it either could not overcome the hydraulic barrier at the grounding line or, if it did discharge into the ocean, the volume of freshwater generated an insufficient buoyancy forcing to overcome grounding line pumping to initiate a meltwater plume that could then advect to our seaward study site. We expect that any remaining subglacial water, which likely consisted primarily of upstream basal meltwater, accumulated at the grounding line throughout the winter season until air temperatures exceeded freezing the following summer, when surface meltwater drained through the glacier and replenished this reservoir. Extensive winter subglacial water has been observed beneath a land‐terminating glacier system in southwest Greenland in April (Chu et al., 2016), as well as weak discharge of this water in February (Pitcher et al., 2020). While the Petermann Gletscher environmental setting differs from these glaciers (marine‐terminating, larger drainage basin, lower air temperatures), our results nonetheless provide another example of SR discharge well after the end of the summer melt season.

## 6 Conclusion

Our results reveal how variability in under‐ice current speeds dictates the rate at which Petermann Gletscher Ice Shelf (PGIS) melts at a location in its central basal channel. High melt rates occurred periodically at our study site over the 3 months following the 2015 summer season, but ended afterward. These high melt rates aligned with estimates of swift under‐ice currents that resulted from the discharge of buoyant subglacial runoff across the glacier's grounding line into the ocean. The flow initiated by this freshwater source efficiently mixed the relatively warm and saline seawater in the underlying ocean mixed layer across the stratified boundary layer, providing a source of heat to melt the ice base. When placed into the larger context of atmospheric warming in northern Greenland (Orsi et al., 2017), these daily to seasonal variations in current speeds that relate to summer surface meltwater production could have a profound effect on the long‐term melt rate beneath PGIS, and could play an integral role in the continued thinning of the ice shelf (Münchow et al., 2014). The combination of warm ocean conditions and swift buoyant currents from subglacial runoff were implicated in the collapse of major Greenland outlet glacier Jakobshavn Isbrae's ice shelf (Holland et al., 2008; Motyka et al., 2011). Two consecutive large calving events in 2010 and 2012 reduced the length of PGIS to an extent that had not yet been observed in recorded history (Münchow et al., 2014), and converging rifts at ∼40 km from the grounding line signal the imminent removal of another large section of the ice shelf (Rückamp et al., 2019). This sustained retreat highlights the relationship between stronger basal melting and destabilization of another Greenland ice shelf. Future changes to the subglacial runoff flux beneath the ice shelf will dictate its stability, with increased flux likely leading to the eventual removal of PGIS (Reilly et al., 2019) and accelerated ice discharge from the Greenland ice sheet (Hill et al., 2018).

## Data Availability

Under‐ice hydrographic profile and mooring data are available in the Arctic Data Center at https://doi.org/10.18739/A22J6846K and https://doi.org/10.18739/A2PC2T86B, respectively. Discovery Harbor pressure measurements are also archived in the Arctic Data Center (at https://doi.org/10.18739/A2MZ3J). Operation Icebridge ice penetrating radar data can be accessed online (at https://data.cresis.ku.edu/data/rds). ApRES basal melt rates are available in the Arctic Data Center at https://doi.org/10.18739/A2T14TQ57.

## Appendix A Current Speeds Required to Form a Tidal Front

### A.1

We investigate the likelihood of a grounding zone tidal front using Equation 16 of Holland (2008) or Equation 3 of MacAyeal (1984):

(A1) $$q_{c}=\frac{4\alpha C_{D}\mu U^{3}}{\beta gS_{A}D},$$

where *q*_*c*_ denotes a critical one dimensional buoyancy flux (speed) when tidal current speeds (*U*) erode stratification to fully mix the water column. In Equation A1, *α* = 0.0075 is the proportion of turbulence used in destratifying the water column, $C_{D}=0.007$ is the drag coefficient derived in section 3.2, *μ* = 2 represents the temporal pattern of tidal speed (Holland, 2008), $\beta =8\times 10^{-4}$ g kg^−1^ is the haline contraction coefficient, g=9.81 m s^−1^ is gravitational acceleration, $S_{A}=34.93$ g kg^−1^ is the salinity of Atlantic Water at the grounding line, and *D* indicates water column thickness.

We assume that SR discharges from the upstream subglacial conduit into a shallow grounding zone water column that is encompassed by the central ice shelf basal channel. We attain values for *q*_*c*_ by dividing the SR flux for the propagating front of each meltwater pulse by a triangular area of 850 m width and varying height between 5 and 30 m, representative of a range of water column thicknesses. As *q*_*c*_ diminishes with an increasingly thick water column, we can thus solve for the constant tidal current speed necessary to trap the flux of each meltwater pulse (Table A1).

**Table A1:** Tidal Currents (*U*) Necessary to Create a Tidal Front, Considering Various Grounding Zone Buoyancy Fluxes (*q*_*c*_) Associated With Meltwater Pulses

Pulse	*q*_*c*_ (m s^−1^)	*U* (m s^−1^)
1	<0.001–0.02	63
2	<0.001–0.01	40
3	<0.001–0.02	63
4	<0.001–0.01	34
5	<0.001	12
6	<0.001–0.04	124

*Note.* The range in *q*_*c*_ represents variability in the one‐dimensional bouyancy fluxes with over a water column thickness range of 5 to 40 m.

## References

[jgrc24212-bib-0001] Adusumilli, S. , Fricker, H. A. , Medley, B. , Padman, L. , & Siegfried, M. R. (2020). Interannual variations in meltwater input to the Southern Ocean from Antarctic ice shelves. Nature Geoscience, 13(9), 616–620. 10.1038/s41561-020-0616-z PMC7500482 32952606

[jgrc24212-bib-0002] Bamber, J. L. , Siegert, M. J. , Griggs, J. A. , Marshall, S. J. , & Spada, G. (2013). Paleofluvial mega‐canyon beneath the central Greenland ice sheet. Science, 341(6149), 997–999. 23990558 10.1126/science.1239794

[jgrc24212-bib-0003] Begeman, C. B. , Tulaczyk, S. M. , Marsh, O. J. , Mikucki, J. A. , Stanton, T. P. , Hodson, T. O. , Siegfried, M. R. , Powell, R. D. , Christianson, K. , & King, M. A. (2018). Ocean stratification and low melt rates at the Ross Ice Shelf grounding zone. Journal of Geophysical Research: Oceans, 123, 7438–7452. 10.1029/2018JC013987

[jgrc24212-bib-0004] Bell, R. E. , Tinto, K. , Das, I. , Wolovick, M. , Chu, W. , Creyts, T. T. , Frearson, N. , Abdi, A. , & Paden, J. D. (2014). Deformation, warming and softening of Greenland's ice by refreezing meltwater. Nature Geoscience, 7(7), 497–502.

[jgrc24212-bib-0005] Cai, C. , Rignot, E. , Menemenlis, D. , & Nakayama, Y. (2017). Observations and modeling of ocean‐induced melt beneath Petermann Glacier Ice Shelf in northwestern Greenland. Geophysical Research Letters, 44, 8396–8403. 10.1002/2017GL073711

[jgrc24212-bib-0006] Chambers, C. , Greve, R. , Altena, B. , & Lefeuvre, P.‐M. (2019). On the possibility of a long subglacial river under the north Greenland ice sheet. The Cryosphere Discussions, 2019, 1–21. https://www.the‐cryosphere‐discuss.net/tc‐2019‐141/

[jgrc24212-bib-0007] Chu, W. , Schroeder, D. M. , Seroussi, H. , Creyts, T. T. , Palmer, S. J. , & Bell, R. E. (2016). Extensive winter subglacial water storage beneath the Greenland Ice Sheet. Geophysical Research Letters, 43, 12–484. 10.1002/2016GL071538

[jgrc24212-bib-0008] Davis, P. E. D. , Johnson, H. L. , & Melling, H. (2019). Propagation and vertical structure of the tidal flow in Nares Strait. Journal of Geophysical Research: Oceans, 124, 281–301. 10.1029/2018JC014122

[jgrc24212-bib-0009] Dow, C. F. , Lee, W. S. , Greenbaum, J. S. , Greene, C. A. , Blankenship, D. D. , Poinar, K. , Forrest, A. L. , Young, D. A. , & Zappa, C. J. (2018). Basal channels drive active surface hydrology and transverse ice shelf fracture. Science Advances, 4(6), eaao7212. 29928691 10.1126/sciadv.aao7212 PMC6007161

[jgrc24212-bib-0010] Dupont, T. K. , & Alley, R. B. (2005). Assessment of the importance of ice‐shelf buttressing to ice‐sheet flow. Geophysical Research Letters, 32, L04503 10.1029/2004GL022024

[jgrc24212-bib-0011] Enderlin, E. M. , Howat, I. M. , Jeong, S. , Noh, M.‐J. , Angelen, J. H. , & Broeke, M. R. (2014). An improved mass budget for the Greenland ice sheet. Geophysical Research Letters, 41, 866–872. 10.1002/2013GL059010

[jgrc24212-bib-0012] Fearnhead, P. G. (1975). On the formation of fronts by tidal mixing around the British Isles. Deep sea research, 22(5), 311–321. 10.1016/0011-7471(75)90072-8

[jgrc24212-bib-0013] Gill, A. E. (1982). Atmosphere‐ocean dynamics, International Geophysics Series (Vol. 30). New York: Academic Press.

[jgrc24212-bib-0014] Hattermann, T. , Nøst, O. A. , Lilly, J. M. , & Smedsrud, L. H. (2012). Two years of oceanic observations below the Fimbul Ice Shelf, Antarctica. Geophysical Research Letters, 39, L12605 10.1029/2012GL051012

[jgrc24212-bib-0015] Herraiz‐Borreguero, L. , Allison, I. , Craven, M. , Nicholls, K. W. , & Rosenberg, M. A. (2013). Ice shelf/ocean interactions under the Amery Ice Shelf: Seasonal variability and its effect on marine ice formation. Journal of Geophysical Research: Oceans, 118, 7117–7131. 10.1002/2013JC009158

[jgrc24212-bib-0016] Hill, E. A. , Carr, J. R. , Stokes, C. R. , & Gudmundsson, H. (2018). Dynamic changes in outlet glaciers in northern Greenland from 1948 to 2015. The Cryosphere, 12(10), 3243–3263.

[jgrc24212-bib-0017] Hill, E. A. , Gudmundsson, G. H. , Carr, J. R. , & Stokes, C. R. (2018). Velocity response of Petermann Glacier, northwest Greenland to past and future calving events. The Cryosphere, 12(12), 3907–3921.

[jgrc24212-bib-0018] Hogg, A. E. , Shepherd, A. , Gourmelen, N. , & Engdahl, M. (2016). Grounding line migration from 1992 to 2011 on Petermann Glacier, North‐West Greenland. Journal of Glaciology, 62(236), 1104–1114.

[jgrc24212-bib-0019] Holland, P. R. (2008). A model of tidally dominated ocean processes near ice shelf grounding lines. Journal of Geophysical Research, 113, C11002 10.1029/2007JC004576

[jgrc24212-bib-0020] Holland, D. M. , & Jenkins, A. (1999). Modeling thermodynamic ice–ocean interactions at the base of an ice shelf. Journal of Physical Oceanography, 29(8), 1787–1800.

[jgrc24212-bib-0021] Holland, D. M. , Thomas, R. H. , De Young, B. , Ribergaard, M. H. , & Lyberth, B. (2008). Acceleration of Jakobshavn Isbrae triggered by warm subsurface ocean waters. Nature geoscience, 1(10), 659–664.

[jgrc24212-bib-0022] Jackson, R. H. , Lentz, S. J. , & Straneo, F. (2018). The dynamics of shelf forcing in Greenlandic fjords. Journal of Physical Oceanography, 48(11), 2799–2827.

[jgrc24212-bib-0023] Jackson, R. H. , Straneo, F. , & Sutherland, D. A. (2014). Externally forced fluctuations in ocean temperature at Greenland glaciers in non‐summer months. Nature Geoscience, 7(7), 503.

[jgrc24212-bib-0024] Jakobsson, M. , Hogan, K. A. , Mayer, L. A. , Mix, A. , Jennings, A. , Stoner, J. , Eriksson, B. , Jerram, K. , Mohammad, R. , Pearce, C. , & Reilly, B. (2018). The Holocene retreat dynamics and stability of Petermann Glacier in northwest Greenland. Nature Communications, 9(1), 2104. 10.1038/s41467-018-04573-2 PMC5974188 29844384

[jgrc24212-bib-0025] Jenkins, A. (1991). A one‐dimensional model of ice shelf‐ocean interaction. Journal of Geophysical Research, 96(C11), 20,671–20,677.

[jgrc24212-bib-0026] Jenkins, A. (2011). Convection‐driven melting near the grounding lines of ice shelves and tidewater glaciers. Journal of Physical Oceanography, 41(12), 2279–2294.

[jgrc24212-bib-0027] Jenkins, A. , Nicholls, K. W. , & Corr, H. ughF. J. (2010). Observation and parameterization of ablation at the base of Ronne Ice Shelf, Antarctica. Journal of Physical Oceanography, 40(10), 2298–2312.

[jgrc24212-bib-0028] Johnson, H. L. , Münchow, A. , Falkner, K. K. , & Melling, H. (2011). Ocean circulation and properties in Petermann Fjord, Greenland. Journal of Geophysical Research, 116, C01003 10.1029/2010JC006519

[jgrc24212-bib-0029] Joughin, I. R. , Smith, B. E. , Howat, I. M. , Scambos, T. , & Moon, T. (2010). Greenland flow variability from ice‐sheet‐wide velocity mapping. Journal of Glaciology, 56(197), 415–430.

[jgrc24212-bib-0030] Joughin, I. R. , Tulaczyk, S. , & Engelhardt, H. F. (2003). Basal melt beneath Whillans ice stream and ice streams A and C, West Antarctica. Annals of Glaciology, 36, 257–262.

[jgrc24212-bib-0080] Lindeman, M. R. , Straneo, F. , Wilson, N. J. , Toole, J. M. , Krishfield, R. A. , Beaird, N. L. , Kanzow, T. , & Schaffer, J. (2020). Ocean circulation and variability beneath Nioghalvfjerdsbræ (79 North Glacier) ice tongue. Journal of Geophysical Research: Oceans, 125, e2020JC016091 10.1029/2020JC016091

[jgrc24212-bib-0032] Münchow, A. , & Melling, H. (2008). Ocean current observations from Nares Strait to the west of Greenland: Interannual to tidal variability and forcing. Journal of Marine Research, 66(6), 801–833.

[jgrc24212-bib-0033] Münchow, A. , Padman, L. , & Fricker, H. A. (2014). Interannual changes of the floating ice shelf of Petermann Gletscher, North Greenland, from 2000 to 2012. Journal of Glaciology, 60(221), 489–499.

[jgrc24212-bib-0034] Münchow, A. , Padman, L. , Washam, P. , & Nicholls, K. W. (2016). The ice shelf of Petermann Gletscher, North Greenland, and its connection to the Arctic and Atlantic Oceans. Oceanography, 29, 84–95.

[jgrc24212-bib-0035] MacAyeal, D. R. (1984). Thermohaline circulation below the Ross Ice Shelf: A consequence of tidally induced vertical mixing and basal melting. Journal of Geophysical Research, 89(C1), 597–606.

[jgrc24212-bib-0036] Mack, S. L. , Dinniman, M. S. , Klinck, J. M. , McGillicuddy Jr, D. J. , & Padman, L. (2019). Modeling ocean eddies on Antarctica's cold water continental shelves and their effects on ice shelf basal melting. Journal of Geophysical Research: Oceans, 124, 5067–5084. 10.1029/2018JC014688

[jgrc24212-bib-0037] Makinson, K. , & Nicholls, K. W. (1999). Modeling tidal currents beneath Filchner‐Ronne Ice Shelf and on the adjacent continental shelf: Their effect on mixing and transport. Journal of Geophysical Research, 104(C6), 13,449–13,465.

[jgrc24212-bib-0038] Makinson, K. , Schröder, M. , & Østerhus, S. (2006). Effect of critical latitude and seasonal stratification on tidal current profiles along Ronne Ice Front, Antarctica. Journal of Geophysical Research, 111, C03022 10.1029/2005JC003062

[jgrc24212-bib-0039] McPhee, M. G. (1979). The effect of the oceanic boundary layer on the mean drift of pack ice: Application of a simple model. Journal of Physical Oceanography, 9(2), 388–400.

[jgrc24212-bib-0040] McPhee, M. G. , Maykut, G. A. , & Morison, J. H. (1987). Dynamics and thermodynamics of the ice/upper ocean system in the marginal ice zone of the Greenland Sea. Journal of Geophysical Research, 92(C7), 7017–7031.

[jgrc24212-bib-0041] Motyka, R. J. , Truffer, M. , Fahnestock, M. , Mortensen, J. , Rysgaard, S. , & Howat, I. (2011). Submarine melting of the 1985 Jakobshavn Isbræ floating tongue and the triggering of the current retreat. Journal of Geophysical Research, 116, F01007 10.1029/2009JF001632

[jgrc24212-bib-0042] Mouginot, J. , & Rignot, E. (2019). Glacier catchments/basins for the Greenland Ice Sheet, UC Irvine, Dataset. 10.7280/D1WT11

[jgrc24212-bib-0043] Mouginot, J. , Rignot, E. , Bjørk, A. A. , van den Broeke, M. , Millan, R. , Morlighem, M. , Noël, B. , Scheuchl, B. , & Wood, M. (2019). Forty‐six years of Greenland Ice Sheet mass balance from 1972 to 2018. Proceedings of the National Academy of Sciences, 116(19), 9239–9244. 10.1073/pnas.1904242116 PMC6511040 31010924

[jgrc24212-bib-0044] Nicholls, K. W. , Corr, H. ughF. J. , Stewart, C. L. , Lok, L. B. , Brennan, P. V. , & Vaughan, D. G. (2015). A ground‐based radar for measuring vertical strain rates and time‐varying basal melt rates in ice sheets and shelves. Journal of Glaciology, 61(230), 1079–1087.

[jgrc24212-bib-0045] Nicholls, K. W. , & Østerhus, S. (2004). Interannual variability and ventilation timescales in the ocean cavity beneath Filchner‐Ronne Ice Shelf, Antarctica. Journal of Geophysical Research, 109, C04014 10.1029/2003JC002149

[jgrc24212-bib-0046] Orsi, A. J. , Kawamura, K. , Masson‐Delmotte, V. , Fettweis, X. , Box, J. E. , Dahl‐Jensen, D. , Clow, G. D. , Landais, A. , & Severinghaus, J. P. (2017). The recent warming trend in North Greenland. Geophysical Research Letters, 44, 6235–6243. 10.1002/2016GL072212

[jgrc24212-bib-0047] Padman, L. , Siegfried, M. R. , & Fricker, H. A. (2018). Ocean tide influences on the Antarctic and Greenland Ice Sheets. Reviews of Geophysics, 56(1), 142–184.

[jgrc24212-bib-0048] Pitcher, L. H. , Smith, L. C. , Gleason, C. J. , Miège, C. , Ryan, J. C. , Hagedorn, B. , van As, D. , Chu, W. , & Forster, R. R. (2020). Direct observation of winter meltwater drainage from the Greenland Ice Sheet. Geophysical Research Letters, 47, e2019GL086521 10.1029/2019GL086521

[jgrc24212-bib-0049] Pritchard, H. D. , Ligtenberg, S. R. M. , Fricker, H. A. , Vaughan, D. G. , Van den Broeke, M. R. , & Padman, L. (2012). Antarctic ice‐sheet loss driven by basal melting of ice shelves. Nature, 484(7395), 502. 22538614 10.1038/nature10968

[jgrc24212-bib-0050] Rückamp, M. , Neckel, N. , Berger, S. , Humbert, A. , & Helm, V. (2019). Calving induced speedup of Petermann Glacier. Journal of Geophysical Research: Earth Surface, 124, 216–228. 10.1029/2018JF004775

[jgrc24212-bib-0051] Reilly, B. T. , Stoner, J. S. , Mix, A. C. , Walczak, M. H. , Jennings, A. , Jakobsson, M. , Dyke, L. , Glueder, A. , Nicholls, K. , & Hogan, K. A. (2019). Holocene break‐up and reestablishment of the Petermann Ice Tongue, Northwest Greenland. Quaternary Science Reviews, 218, 322–342.

[jgrc24212-bib-0052] Rhines, P. (1970). Edge‐, bottom‐, and Rossby waves in a rotating stratified fluid. Geophysical Fluid Dynamics, 1(3–4), 273–302. 10.1080/03091927009365776

[jgrc24212-bib-0053] Rignot, E. , Gogineni, S. , Joughin, I. , & Krabill, W. (2001). Contribution to the glaciology of northern Greenland from satellite radar interferometry. Journal of Geophysical Research, 106(D24), 34,007–34,019.

[jgrc24212-bib-0054] Rignot, E. , & Jacobs, S. S. (2002). Rapid bottom melting widespread near Antarctic ice sheet grounding lines. Science, 296(5575), 2020–2023. 12065835 10.1126/science.1070942

[jgrc24212-bib-0055] Rignot, E. , Jacobs, S. , Mouginot, J. , & Scheuchl, B. (2013). Ice‐shelf melting around Antarctica. Science, 341(6143), 266–270. 23765278 10.1126/science.1235798

[jgrc24212-bib-0056] Rignot, E. , & Kanagaratnam, P. (2006). Changes in the velocity structure of the Greenland Ice Sheet. Science, 311(5763), 986–990. 16484490 10.1126/science.1121381

[jgrc24212-bib-0057] Rignot, E. , Mouginot, J. , Morlighem, M. , Seroussi, H. , & Scheuchl, B. (2014). Widespread, rapid grounding line retreat of Pine Island, Thwaites, Smith, and Kohler glaciers, West Antarctica, from 1992 to 2011. Geophysical Research Letters, 41, 3502–3509. 10.1002/2014GL060140

[jgrc24212-bib-0058] Rignot, E. , & Steffen, K. (2008). Channelized bottom melting and stability of floating ice shelves. Geophysical Research Letters, 35, L02503 10.1029/2007GL031765

[jgrc24212-bib-0059] Robertson, R. , Dong, J. , & Hartlipp, P. (2017). Diurnal critical latitude and the latitude dependence of internal tides, internal waves, and mixing based on Barcoo Seamount. Journal of Geophysical Research: Oceans, 122, 7838–7866. 10.1002/2016JC012591

[jgrc24212-bib-0060] Rosier, S. , & Gudmundsson, H. (2020). Exploring mechanisms responsible for tidal modulation in flow of the Filchner–Ronne Ice Shelf. The Cryosphere, 14(1), 17–37.

[jgrc24212-bib-0061] Sayag, R. , & Worster, M. G. (2013). Elastic dynamics and tidal migration of grounding lines modify subglacial lubrication and melting. Geophysical Research Letters, 40, 5877–5881. 10.1002/2013GL057942

[jgrc24212-bib-0062] Shepherd, A. , Ivins, E. , Rignot, E. , Smith, B. , Van Den Broeke, M. , Velicogna, I. , Whitehouse, P. , Briggs, K. , Joughin, I. , Krinner, G. , & Nowicki, S. (2018). Mass balance of the Antarctic Ice Sheet from 1992 to 2017. Nature, 558, 219–222. 29899482 10.1038/s41586-018-0179-y

[jgrc24212-bib-0063] Simpson, J. H. , & Hunter, J. R. (1974). Fronts in the Irish Sea. Nature, 250(5465), 404.

[jgrc24212-bib-0064] Smith, B. , Fricker, H. A. , Gardner, A. S. , Medley, B. , Nilsson, J. , Paolo, F. S. , Holschuh, N. , Adusumilli, S. , Brunt, K. , Csatho, B. , & Harbeck, K. (2020). Pervasive ice sheet mass loss reflects competing ocean and atmosphere processes. Science, 368(6496), 1239–1242. 32354841 10.1126/science.aaz5845

[jgrc24212-bib-0065] Steele, M. , Mellor, G. L. , & Mcphee, M. G. (1989). Role of the molecular sublayer in the melting or freezing of sea ice. Journal of Physical Oceanography, 19(1), 139–147.

[jgrc24212-bib-0066] Stewart, C. L. , Christoffersen, P. , Nicholls, K. W. , Williams, M. J. M. , & Dowdeswell, J. A. (2019). Basal melting of Ross Ice Shelf from solar heat absorption in an ice‐front polynya. Nature Geoscience, 12(6), 435.

[jgrc24212-bib-0067] Sun, S. , Hattermann, T. , Pattyn, F. , Nicholls, K. W. , Drews, R. , & Berger, S. (2019). Topographic shelf waves control seasonal melting near Antarctic ice shelf grounding lines. Geophysical Research Letters, 46, 9824–9832. 10.1029/2019GL083881

[jgrc24212-bib-0068] Thomas, R. H. , & Bentley, C. R. (1978). A model for Holocene retreat of the West Antarctic ice sheet. Quaternary Research, 10(2), 150–170.

[jgrc24212-bib-0069] Vaňková, I. , Nicholls, K. W. , Corr, H. F. J. , Makinson, K. , & Brennan, P. V. (2020). Observations of tidal melt and vertical strain at the Filchner‐Ronne Ice Shelf, Antarctica. Journal of Geophysical Research: Earth Surface, 125, e2019JF005280 10.1029/2019JF005280

[jgrc24212-bib-0070] van den Broeke, M. , Bamber, J. , Ettema, J. , Rignot, E. , Schrama, E. , van de Berg, W. J. , van Meijgaard, E. , Velicogna, I. , & Wouters, B. (2009). Partitioning recent Greenland mass loss. Science, 326(5955), 984–986. 19965509 10.1126/science.1178176

[jgrc24212-bib-0071] Walker, R. T. , Parizek, B. R. , Alley, R. B. , Anandakrishnan, S. , Riverman, K. L. , & Christianson, K. (2013). Ice‐shelf tidal flexure and subglacial pressure variations. Earth and Planetary Science Letters, 361, 422–428.

[jgrc24212-bib-0072] Washam, P. , Münchow, A. , & Nicholls, K. W. (2018). A decade of ocean changes impacting the ice shelf of Petermann Gletscher, Greenland. Journal of Physical Oceanography, 48, 2477–2493.

[jgrc24212-bib-0073] Washam, P. , Nicholls, K. W. , Münchow, A. , & Padman, L. (2019). Summer surface melt thins Petermann Gletscher Ice Shelf by enhancing channelized basal melt. Journal of Glaciology, 65(252), 662–674. 10.1017/jog.2019.43 PMC6662214 31359886

[jgrc24212-bib-0074] Webber, BGM , Heywood, K. J. , Stevens, D. P. , Dutrieux, P. , Abrahamsen, E. P. , Jenkins, A. , Jacobs, S. S. , Ha, H. K. , Lee, S. H. , & Kim, T. W. (2017). Mechanisms driving variability in the ocean forcing of Pine Island Glacier. Nature Communications, 8(1), 1–8. 10.1038/ncomms14507 PMC5321733 28211473

[jgrc24212-bib-0075] Wilson, N. , Straneo, F. , Heimbach, P. , & Cenedese, C. (2017). Satellite‐derived submarine melt rates and mass balance (2011–2015) for Greenland's largest remaining ice tongues. The Cryosphere Discussions, 11, 2773–2782.

[jgrc24212-bib-0076] Xu, Y. , Rignot, E. , Fenty, I. , Mememenlis, D. , & Flexas, M. M. (2013). Subaqueous melting of Store Glacier, west Greenland from three‐dimensional, high‐resolution numerical modeling and ocean observations. Geophysical Research Letters, 40, 4648–4653. 10.1002/grl.50825

